# Amyloid Deposition in Transplanted Human Pancreatic Islets: A Conceivable Cause of Their Long-Term Failure

**DOI:** 10.1155/2008/562985

**Published:** 2009-03-05

**Authors:** Arne Andersson, Sara Bohman, L. A. Håkan Borg, Johan F. Paulsson, Sebastian W. Schultz, Gunilla T. Westermark, Per Westermark

**Affiliations:** ^1^Department of Medical Cell Biology, Uppsala University, 751 23 Uppsala, Sweden; ^2^Division of Cell Biology, Diabetes Research Centre, Linköping University, 581 83 Linköping, Sweden; ^3^Department Genetics and Pathology, Uppsala University, 751 85 Uppsala, Sweden

## Abstract

Following the encouraging report of the Edmonton group, there was a rejuvenation of the islet transplantation field. After that, more pessimistic views spread when long-term results of the clinical outcome were published. A progressive loss of the *β*-cell function meant that almost all patients were back on insulin therapy after 5 years. More than 10 years ago, we demonstrated that amyloid deposits rapidly formed in human islets and in mouse islets transgenic for human IAPP when grafted into nude mice. It is, therefore, conceivable to consider amyloid formation as one potential candidate for the long-term failure. The present paper reviews attempts in our laboratories to elucidate the dynamics of and mechanisms behind the formation of amyloid in transplanted islets with special emphasis on the impact of long-term hyperglycemia.

## 1. INTRODUCTION

The discovery of insulin in the early 1920s greatly improved the prognosis for type 1 diabetes patients and by such means patients with diabetes could survive a previously fatal disease. Because of the substantial improvements in insulin therapy, most patients nowadays can handle their treatment themselves and risks for the crippling long-term complications have become extensively reduced. This, however, requires strict blood glucose control and life style restrictions. These latter insufficiencies of the present treatment together with the fact that a subgroup of patients is still disturbed by frequent hypoglycaemic attacks have meant that there is considerable interest in pancreatic islet transplantation. For long replacement of the destroyed *β*-cells in type 1 diabetes with new *β*-cells, this has attracted much attention. Paul Lacy's pioneering work with his collagenase-based method for rat islet isolation paved the way for islet transplantation experiments. Clinical trials were carried out in the 80s and 90s but only about 10% of islet recipients achieved normoglycemia without insulin therapy. However, in their report in the year 2000 James Shapiro et al. reported a handful of diabetes patients all of whom became normoglycemic after two or three intraportal implantations of noncultured human islets [1]. Given a steroid-free immunosuppression, these patients remained off insulin for at least one year. In an international trial of this so-called Edmonton protocol, 36 subjects with type 1 diabetes underwent this type of treatment at nine international sites [2]. While 16 of them (44%) were insulin free after one year only 5 (14%) remained so after one more year. It was concluded that there was a progressive loss of islet function in most subjects, who had all become insulin independent initially.

For long, it has been postulated that long-term hyperglycemia might influence *β*-cell function in a negative way. Numerous in vitro and in vivo studies have indicated that so is the case but the molecular mechanisms are still unclear. We, therefore, found it conceivable to consider amyloid formation as one potential candidate. This paper reviews attempts in our laboratory to elucidate the fate of transplanted human islets with a special view on their morphology and function and especially so under influence of prolonged hyperglycemic stress.

## 2. ISLET AMYLOID POLYPEPTIDE AND ISLET AMYLOID

Although islet amyloid was discovered already in 1901 [3, 4], its impact in the pathogenesis of type 2 diabetes has been questioned for a long period of time. However, there are several lines of evidence for the importance of the amyloid formation for the *β*-cell lesion in type 2 diabetes (for reviews, see [5, 6]). The exact mechanisms are still not very well understood but aggregated IAPP is toxic to *β*-cells [7, 8].

**Figure 1:**
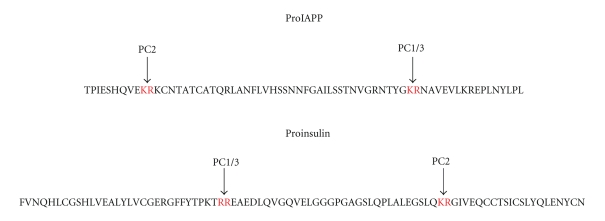
Processing at double basic amino acid residues of proinsulin and proIAPP by the prohormone convertases PC 1/3 and PC2.

IAPP was discovered by purification and analysis of amyloid, first from a human insulinoma [9, 10] and later from islets of Langerhans [11, 12]. The same peptide was found to form amyloid in apes [13, 14] and cats [11, 15]. Human IAPP is a 37-amino acid residue peptide, expressed as a prepromolecule. After removal of the signal peptide, the 67-amino acid propeptide is further processed at two double basic residues by the prohormone convertases PC2 and PC1/3 which remove two short peptides N- and C-terminally (Figure 1). The remaining peptide is C-terminally amidated and there is a disulfide bridge between residues 2 and 7.

IAPP is expressed by *β*-cells and is stored and released together with insulin. IAPP is very aggregation-prone in vitro and rapidly forms amyloid-like fibrils. This does not normally happen in vivo, where there must be mechanisms which hinder this. Binding to insulin may be such a mechanism [16, 17]. However, it is not understood why IAPP aggregates into amyloid in conjunction with type 2 diabetes. Experiments with transgenic mice, overexpressing human IAPP, clearly indicate that an increased production of IAPP is not the single explanation but that other factors must contribute.

### 2.1. Transgenic animals overexpressing human IAPP

Mice and rats do not develop islet amyloid, depending on differences in the IAPP sequence. Proline residues in the amyloid-forming core of IAPP abolish the fibril formation in both species [18]. Several groups have, therefore, created transgenic mouse lines expressing human IAPP under regulation of an insulin promoter. In spite of overexpression of human IAPP, islet amyloid generally does not develop. However, amyloid does appear when such animals are fed a diet high in fat [19, 20] or are crossed with ob/ob [21] or agouti [22] mice. We are working with a mouse line, overexpressing human IAPP behind rat insulin 1 promoter but devoid of mouse IAPP. Animals of this strain do not spontaneously develop islet amyloid at any age but in male mice, when fed a diet with high content of fat, amyloid deposits occur at an age of 11 months [20]. The amyloid is mainly found extracellularly but intracellular deposits do occur [23].

### 2.2. Amyloid development in cultured human and transgenicmouse islets

Interestingly, islets isolated from our transgenic mouse strain develop amyloid deposits rapidly when cultured in vitro [24]. A similar experience was obtained with another human IAPP transgenic mouse strain [25]. Furthermore, in contrast to what is found in islets in type 2 diabetes, where the amyloid is extracellular [26], intracellular aggregation of IAPP initially takes place in cultured human islets [27]. The exact compartmental position has been difficult to determine but is probably the endoplasmic reticulum or Golgi apparatus [28].

### 2.3. Aberrant processing and amyloid formation

 There is evidence that the intracellular amyloid contains proIAPP and a defect processing of this precursor to mature IAPP may play a role in the pathogenesis of amyloid formation [23]. *β*-Cell stress that occurs in the initial phase of type 2 diabetes results in a disproportional secretion of unprocessed or partially processed proinsulin (des 32-33 C-peptide-A-chain fragment) [29]. This shift can mirror an increase in granule turnover, or, perhaps more interestingly is a sign of incomplete processing due to convertase deficiency. Also the prohormone convertases PC 1/3 and PC2 themselves must undergo cleavage to become active, and therefore, aberrant activation of convertases can lead to incomplete processing. Proinsulin is processed by PC1/3 at the B-chain/C-peptide junction followed by PC2 cleavage at the C-peptide/A-chain junction while PC1/3 and PC2 processing of proIAPP results in the removal of the C-terminal and N-terminal flanking peptides, respectively [30]. In vitro, IAPP is one of the more aggregation-prone amyloid peptides known and insulin has been shown to exert a concentration-dependent inhibitory effect on IAPP fibril formation at neutral pH. We have produced human IAPP and partially processed proIAPP, lacking the C-terminal flanking peptide (NIAPP) with recombinant technology [31]. In the following, previously unpublished study, IAPP or NIAPP (20 *μ*M) and insulin (40 *μ*M) were dissolved in 25 mM phosphate buffer with 50 mM glycine at pH 7 and pH 5.2. Aliquots were analyzed for the presence of amyloid fibrils after Congo red staining. We conferred our earlier findings that addition of insulin to IAPP delays fibril formation at pH 7.0 and this was also true for NIAPP. However, at pH 5.2 the fibril formation was triggered for both IAPP and NIAPP. Semiquantitative analysis of amyloid amount, based on Congo red staining and electron microscopical analyses, showed that NIAPP was more prone to form amyloid-like fibrils than mature IAPP. Since both NIAPP and the des 32-33 C-peptide-A-chain proinsulin derivative are expected to appear in the secretory granules as a consequence of reduced PC2 processing, we also expressed des 32-33 C-peptide-A-chain proinsulin. NIAPP and 32-33 C-peptide-A-chain proinsulin were solubilized as described above and mixed 1:1 and 1:4. It was then shown that addition of 32-33 C-peptide-A-chain proinsulin to NIAPP promotes fibril formation. These previously unpublished results show that the intragranular composition of prohormones and processing metabolites is of importance and changes of the equilibrium can be a factor that causes IAPP to aggregate. Transfection of human proIAPP to cell lines missing one or both of the processing enzymes has supported this conclusion since the aberrant processing resulted in increased amyloid formation [32, 33].

## 3. INFLUENCE OF HYPERGLYCEMIA ON GRAFTED HUMAN ISLETS

### 3.1. Electron microscopical appearance

**Figure 2:**
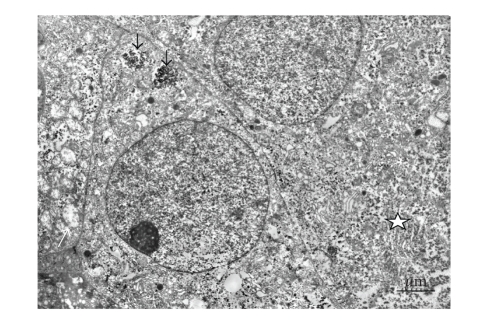
Electron micrograph of human islets transplanted under the kidney capsule of an alloxan-diabetic athymic nude mouse four weeks after implantation. Note the extensive degranulation, the abundant endoplasmic reticulum (star), glycogen particles (black arrows), and swollen mitochondria (white arrow).

In general, the ultrastructure of human islets grafted into normoglycemic mice remains normal 4 weeks after implantation [34]. The *β*-cells are in great majority. A 4-week hyperglycaemic period induces well-known signs of *β*-cell hyperactivity such as marked degranulation and also signs of the development of an abundant rough endoplasmic reticulum (Figure 2). We also observed signs of glycogen particles accumulating in the *β*-cells. These glycogen depositions disappear when transferring the islets to a normoglycemic milieu by curing the recipient by means of implantation of a second islet graft. Interestingly, the mitochondria residing in the hyperglycaemic, noncured recipients are often swollen (Figure 2).

 Taken together, these previous ultrastructural investigations show that the transmission electron microscopical tool is of utmost importance when elucidating the impact of different functional loads put on human islets. Obviously, the knowledge on the classical “hydropic degeneration,” later referred to as “ballooning degeneration” described by Weichelbaum and Stangl, Allen, Toreson, and Lazarus and Volk [35], in reality has become extended by the findings of the glycogen accumulations described above. Likewise, the very early reports on hyalinization of the islets of patients with diabetes by Opie, in 1901 [3], have formed the platform for extensive studies, both morphological and biochemical, on the formation of amyloid deposits (described below).

### 3.2. Functional properties

**Figure 3:**
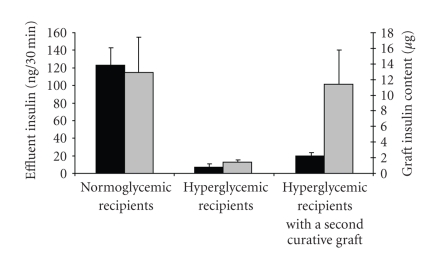
Total insulin secretion in effluent medium collected from human islet graft-bearing kidneys during 30-minute perfusion with 16.7 mM glucose (black bars), and insulin content of renal subcapsular islet grafts (grey bars). Values are means ± SEM.

The ultrastructural findings were corroborated by measurements of the islet graft insulin content (Figure 3). Thus, the high glucose-exposed islet grafts contained about one tenth of the insulin found in the normoglycemic control grafts indicating a parallelism between low insulin content and extensive *β*-cell degranulation. In graft perfusion experiments, where test substances were infused via the renal artery and effluents collected from the ureter and renal vein [36], we found that a high glucose challenge in the test medium increased the insulin concentration of the effluent medium in a biphasic mode when the graft had resided in a normoglycemic recipient not treated with alloxan. Quite in contrast, islet grafts exposed to a high (more than 20 mM) glucose concentration in vivo for 4 weeks displayed a blunted insulin secretion. In fact, the integrated area under the curve, that is, the amount of insulin secreted during the 30-minute stimulation period, was less than 5% of that observed for the control, normoglycemic grafts (Figure 3). Interestingly, this extensively impaired glucose-stimulated insulin secretion was only marginally returned to normal after a 2-week period of normoglycemia effected by a second intrasplenic mouse islet graft (Figure 3). This was despite a nearly total reconstitution of the insulin content of the graft.

In further studies of this defective glucose-induced insulin release of the human islet grafts, we found that also arginine-stimulated secretion was heavily impaired [37]. Neither impaired glucose metabolism nor decreased (pro)insulin biosynthesis could explain the deleterious effects of the diabetic state on human islet graft insulin secretion. It is tempting to speculate that formation of intracellular amyloid deposits might be one hitherto neglected reason for this functional impairment. With our present knowledge, attention should be paid to functional abnormalities also in IAPP biosynthesis and secretion. One process of particular interest in this context might be the enzymatic cleavage of pro-IAPP by the converting enzymes PC 2 and PC 1/3 [38].

## 4. AMYLOID DEPOSITS IN TRANSPLANTED PANCREATIC ISLETS INFLUENCE OF IMPLANTATION SITE, FUNCTIONAL ACTIVITY, AND MICROENCAPSULATION

 In our first report on the rapid deposition of amyloid in human islets transplanted into nude mice, our primary aim was to study the occurrence of IAPP-positive cells in the grafts [39]. Not surprisingly, comparisons of adjacent human islet graft sections stained for insulin and IAPP, respectively, indicated that the antisera stained the same cells. However, while the insulin staining was fairly even, both strongly and weakly labelled cells occurred after staining for IAPP. Interestingly, we found a lower percentage of IAPP-positive cells in the grafts of hyperglycaemic mice, suggesting that the storage of the substance was decreased after hyperglycemia.

By means of Congo red staining, we found amyloid deposits in human islet transplants in six out of eight normoglycaemic and two out of four hyperglycaemic recipients. All these islet grafts had resided under the kidney capsule of the nude mice for no more than two weeks, demonstrating the rapidity of the process. Thus, no amyloid was found in sections of the donor pancreata collected before they were processed for islet isolation. The amyloid deposits were usually multiple and small and located extracellularly but some faintly stained deposits were also found in the cytoplasm of the islet cells.

**Figure 4:**
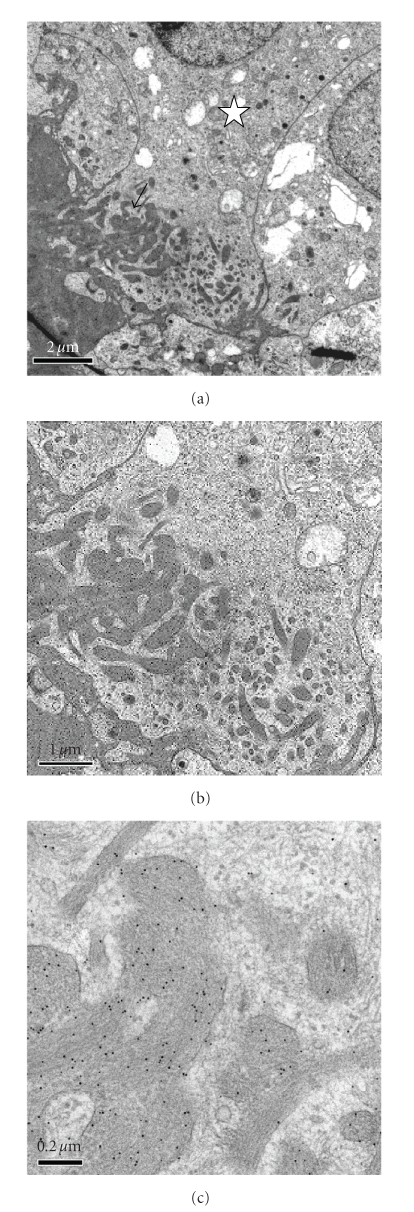
Intra- and extracellular amyloid in an islet graft implanted under the renal capsule of a nude mouse. (a) In the overview, it is seen that the amyloid (arrow) is present in the periphery of degranulated *β*-cells (star). (b) At higher magnification, it is obvious that the amyloid forms a network, presumably due to presence in the endoplasmic reticulum. (c) At high magnification, the fibrillar ultrastructure of the amyloid is evident as well as its specific immunolabelling with antibodies against IAPP, visualized with 10 nm gold particles.

Electron microscopical investigations showed explicitly that IAPP immunoreactivity normally was confined to the secretory granules of the *β*-cells, while *α*- and *δ*-cells were negative. Moreover, as in the light microscopical study, accumulation of amyloid material, strongly labelled with antisera to IAPP, was found in eight of the twelve grafted mice (Figures 4(a) and 4(b)). Large amounts of amyloid fibrils were easily recognized (Figure 4(c)) but sometimes the material also had a granular appearance.

It is worthy of note that in a comparative study elucidating the amyloid deposition in islets of transgenic mice expressing hIAPP and in human islets implanted into nude mice, we found considerable differences [27]. Thus, in human islets amyloid was mainly formed intracellularly (Figure 4), whilst in islets from transgenic mice the amyloid was exclusively deposited extracellularly. Later studies have shown, however, that also in these animals the first amyloid occurs within *β*-cells [38].

 Descriptions of amyloid formation in grafted islets in this paper have all referred to studies using the subcapsular renal space as implantation site. Since essentially all clinical islet transplantations are performed by intraportal infusion, we were interested in investigating intraportally grafted islets as well. Again, nude mice were used as recipients and indeed amyloid exhibiting affinity for Congo red was found in 8 of 9 islet-containing livers (a total of 10 mice were implanted with human islets) [28]. Both quantitatively and qualitatively, the formation of amyloid seemed to occur to the same extent and similarly to that seen in the subcapsularly grafted islets. Separate studies of intrasplenic islet grafts showed that also such islets contained amyloid with the same appearance as in the intraportally implanted human islets.

 While we were unable to demonstrate an effect of hyperglycemia on the amount of amyloid formed in our first study when using both normoglycemic and alloxan-diabetic recipients long-term (14 d) culture of the human islets prior to transplantation seemed to considerably enhance the amyloid formation [28]. At least this was the case in specimens observed for a short-time period—the grafts were evaluated already after 2 weeks. Taken together with results from studies of grafts kept under the kidney capsule for half a year [28] where rather large extracellular deposits were found, it appears that the first amyloid is formed intracellularly and that amyloid at a later stage acts as a nidus for further extracellular deposition. For some reason, however, the process halts and therefore the heavy amyloid deposition as seen in the islets of type 2 diabetes patients never develops. The reasons for this are still unknown but obviously the present experimental model offers unique opportunities for such studies.

**Figure 5:**
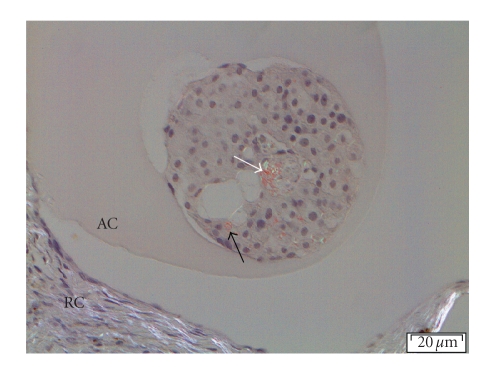
Polarized light microscopic image of a Congo red stained microencapsulated human islet residing in the renal subcapsular space of an athymic nude mouse for four weeks. The black arrow points out amyloid in and outside a normal islet cell, whereas the white arrow indicates amyloid in the central necrotic part of the islet. Surrounding the islet is the alginate capsule (AC), and in the lower part of the image the renal capsule (RC).

One circumstance that might explain the rapid deposition of amyloid in the grafted islets is their fairly low vascular density as compared with the endogenous islets in the pancreas [40]. Such a relative lack of blood vessels providing for an efficient export of the secretory products thus might facilitate the accumulation of IAPP and formation of amyloid. The ultimate test of that hypothesis would be to look for the presence of amyloid deposits in microencapsulated islets, which exemplify a totally nonvascularized islet graft. For that purpose, we encapsulated both human islets and hIAPP transgenic mouse islets in a high-guluronic alginate solution [41]. These capsules were subsequently transplanted into the renal subcapsular space of normoglycemic nude mice [42]. Indeed, preliminary results suggest that encapsulated human islet grafts that were retrieved one month after implantation contained considerable amounts of amyloid (Figure 5). Obviously, under these specific conditions amyloid deposits develop, thus demonstrating that a sustained blood supply is not a prerequisite for their formation. It also seems feasible to use the microencapsulated islets as a tool for more detailed studies of the amyloid formation process under forced circumstances.

## 5. CLINICAL IMPLICATIONS

At present, reports on the pathology of clinically grafted islets are very scarce and to our knowledge amyloid has not been looked for specifically except for our recent study [43]. There are methodological difficulties, one of which might be the fairly long ischemic periods before liver tissue can be harvested. Nevertheless, studies aiming at the localization and characterization of the implanted islets are highly warranted. Since the identification of the amyloid material is often laborious, consultations with groups experienced in this field of research might be desirable. During the final preparation of this manuscript, we published data indeed demonstrating widespread amyloid deposition in clinically transplanted human islets [43]. A patient with type 1 diabetes for more than 35 years died in a myocardial infarction 5 years after the first of three intraportal islet infusions. In almost every second of a total of 89 islets found in the liver tissue blocks, amyloid deposits, most of them being extracellular, were identified. Immunoelectron microscopy demonstrated amyloid fibrils that were positive for antibodies against IAPP. Indeed, these findings highly strengthen the validity of our hypothesis.

## 6. FUTURE PERSPECTIVES

 Long-term results with clinical islet transplantation are fairly discouraging. There is evidence to suggest that this is caused by a progressive loss of the grafted *β*-cells. Knowledge on the nature of that process is, however, meagre. Therefore, the importance of performing necropsies of as many as possible of deceased patients with islet grafts, functioning or nonfunctioning, cannot be enough underlined. Pathologists, experienced in different aspects of islet pathology, including islet amyloidosis, should be consulted when judging the harvested material. By such means, further insights on the nature of the destructive process(es) should be gained.

 As regards, the pathogenic mechanisms of islet amyloidosis, islet transplantation models might offer unique possibilities to study them in more detail. We have very much focussed on the first intracellular IAPP aggregation and the role of proIAPP and proIAPP intermediates in that process. It remains to be established that under circumstances when concentrations of such molecules are high, there is an enhanced amyloid formation in vivo.

In the Edmonton protocol, preparative islet culture was not used perhaps because such manoeuvres might decrease the viability of the isolated islets. Although that view is controversial, it cannot be ruled out that amyloid develops during culture of human islets or mouse islets transgenic for hIAPP. Indeed, there is some evidence in support of that view [28, 44]. However, it has to be proven that such pretransplant deposits indeed stimulate a further and more extensive formation of amyloid in the islets once they have become transplanted.

Finally, it is still an open question as to whether enhanced insulin production, as under hyperglycemic conditions, promotes amyloid growth in the transplanted islets. A general suppression of *β*-cell function by means of insulin treatment or, at least under experimental conditions, drugs like somatostatin and its analogs or diazoxide might be of value to test. In this context, other types of medical intervention against IAPP aggregation should be of interest as well. One such substance is eprodisate, which recently was shown to slow the decline of renal function in patients with AA amyloidosis [45].
